# Beyond asking: Exploring the use of automatic price evaluations to implicitly estimate consumers’ willingness-to-pay

**DOI:** 10.1371/journal.pone.0219251

**Published:** 2019-07-03

**Authors:** Jasper Dezwaef, Emiel Cracco, Jelle Demanet, Timothy Desmet, Marcel Brass

**Affiliations:** 1 Department of Experimental Psychology, Ghent University, Ghent, Belgium; 2 Profacts, De Pinte, Belgium; 3 Faculty of Economic and Social Sciences, Vrije Universiteit Brussel, Brussels, Belgium; 4 Solvay Business School, Brussels, Belgium; 5 Hult International Business School, UG Campus London, London, United Kingdom; Shandong University of Science and Technology, CHINA

## Abstract

Explicit consumers responses are often adverse for the validity of procedures used to estimate consumers’ willingness-to-pay (WTP). This paper investigates if price evaluations occur automatically and to what extent these automatic processes can be used to implicitly estimate consumers’ WTP. An adapted version of the task-rule congruency (TRC) paradigm was used in two studies. Results of the first study provided evidence for the notion that prices are automatically evaluated. However, the used procedure had limitations that restricted its utility as an implicit WTP estimate. The procedure was adjusted, and an additional study was conducted. The results of the second study also indicated that prices were evaluated automatically. Additionally, the procedure used during the second study allowed to explore to what extent the observed TRC effects could be used to implicitly estimate consumers’ WTP. Taken together, these studies provided evidence for the notion that prices are evaluated automatically. Furthermore, the procedure has the potential to be further developed into an implicit estimate of consumers’ WTP.

## Introduction

Methods used to estimate consumers’ willingness-to-pay (WTP) are often based on self-reports and are therefore open to various types of response biases [1,2]. For example, some consumers do not want to share information about their WTP [3], other consumers do not explicitly know what they want to pay [4] or are uncertain about their response [5], and yet others are simply unable to express it [6]. Hence, these procedures often result in inaccurate WTP estimations [7,8].

Willingness-to-pay is a multi-faceted concept defined as the maximum price at which consumers are willing to buy products or services [9,10]. Existing WTP estimation procedures can be divided into direct and indirect methods, both with specific advantages and disadvantages [11]. Direct approaches have the benefit of practicality, low cost, and straightforward analysis. Consumers are merely asked to state if they consider a price fair for a product [12–14] or if they would be willing to pay a specific price for the product [15]. Despite the clear advantages, there are concerns about the manifestation of strategic answering biases of direct methods [16,17]. To address this issue, other methods use indirect procedures based on the assessment of choice behavior to estimate consumers’ WTP. In particular, choice-based conjoint (CBC) analysis is often used to reveal consumers’ indirect WTP [18]. Consumers participating in CBC exercises are strained to make a series of preference judgements regarding a number of products. Analyses of the choices made by participants reveal indirect preference structures, including preferences about the price of the product. These price preferences are then modeled and interpreted as estimates of consumers’ WTP. However, while CBC analysis is a valuable tool to investigate consumers’ decision making, the procedure still depends on stated consumers preferences. Moreover, the application is often cumbrous, and the analysis complex.

Hence, it is clear that pricing practice would benefit from new techniques that obviate problems related to stated consumer responses [19]. More specifically, it would be useful to have estimates of WTP that are not derived from explicit judgments but rather from implicit processes. This idea is not new in consumers research, numerous studies have demonstrated that implicit methods are successful at uncovering consumers’ ‘true’ perceptions [20–22]. However, to the best of our knowledge, this has never been applied to price evaluations. This paper outlines a procedure that proposes to use the reference price of consumers to investigate if prices are evaluated automatically. We assumed that the evaluation of price stimuli, just like the evaluation of other stimuli, can occur immediately, unintentionally and implicitly [23–25].

The reference price was used as a starting point since this concept plays an important role in the construction of consumers’ WTP [26–28]. Consumers use reference prices as a threshold to evaluate the selling price of products, which on its turn impacts subsequent purchase decisions [29–31]. In general, there is consensus that two types of reference prices can be dissociated from one another: external and internal reference prices [32]. While external reference prices refer to the prices of competing brands encountered while shopping [31,32], internal reference prices are prices constructed within the memories of consumers. This internal construct results out of previous encounters with similar products and their prices [33]. Here, we investigate the influence of the internal reference price on automatic price evaluations. We argue that information about this interaction can be used to make implicit WTP estimates. Consequently, this procedure can be used to circumvent problems inherent to WTP estimates based on self-reports. The aim of the present study is to provide ‘proof of principle’, rather than to introduce a method that can be immediately applied.

To test these hypotheses, a paradigm from the literature on the task rule congruency (TRC) effects was adapted. In cognitive psychology the TRC effect is often used to study automatic processes underlying cognitive mechanisms [34–38]. The basic idea is that a specific task-rule introduced in the context of one task affects responding in another task when the same stimuli are used in both tasks [35,38]. For example, if participants have to respond to the identity of a letter in one task (e.g. participants have to press a left key for the letter ‘F’ and a right key for the letter ‘J’) and to the font in another task (e.g. participants have to press a left key for letters in italics and a right key for letters in regular font). Responses are typically faster if both response rules require the same ‘congruent’ response (e.g. F in italics or J in regular) than if they require a different ‘incongruent’ response (J in italics or F in regular). Because the task-rule used in the first task influences responding in the second task, even though it is no longer relevant, this shows that the irrelevant task-rule was automatically processed [37]. We extended the TRC logic to investigate the automatic evaluation of price stimuli.

### Outline of the presented procedure

**Fig 1:**
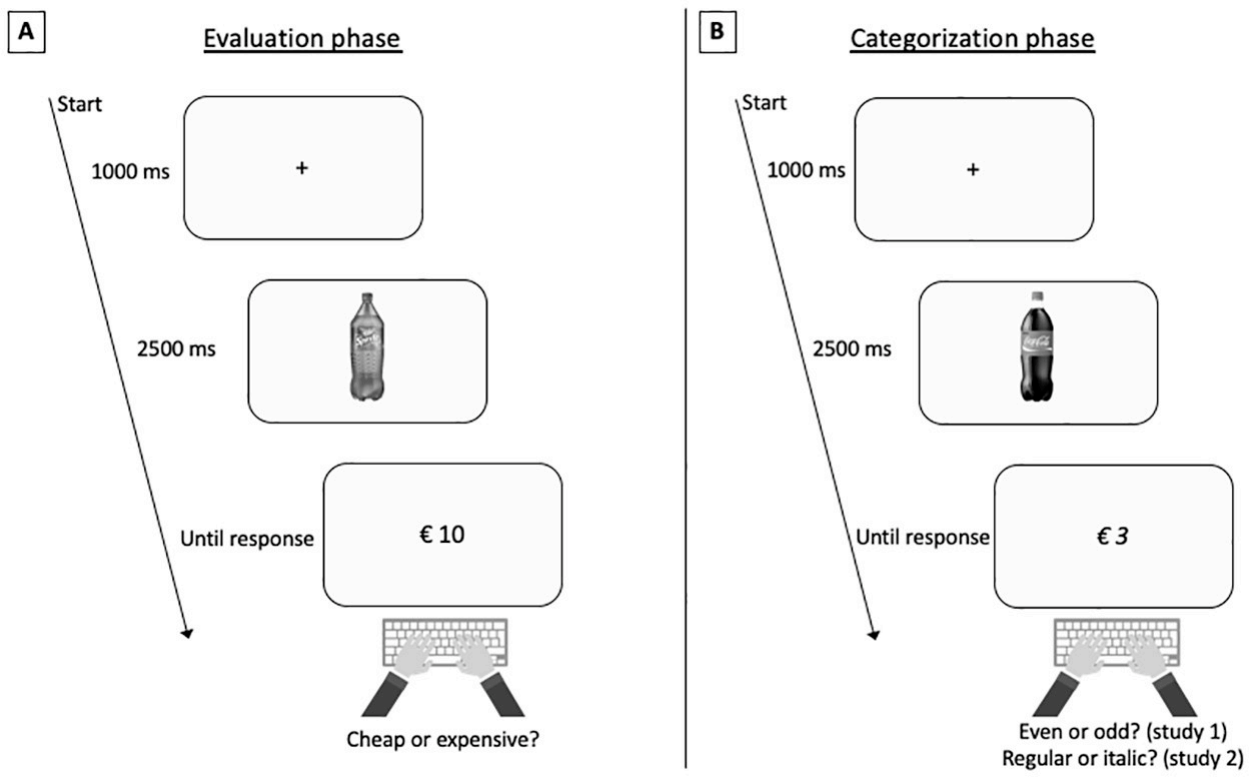
Schematic overview of the trials used during the studies. The evaluation phase (Fig 1 A) started with the presentation of a fixation cross in the middle of the screen. The cross lasted for 1000 ms. When the fixation cross disappeared, the product was presented for 2500 ms. Finally, the product was replaced by the price and participants had to press a key as fast as possible to evaluate the price as ‘cheap’ or ‘expensive’. After the evaluation phase (Fig 1 A) the categorization phase (Fig 1 B) was initiated. The presentation timings of the stimuli were identical to the timings used in the evaluation phase (Fig 1 B). Importantly, the task of the participants changed while the keys used to respond were the same. Now they had to categorize the price as fast as possible based on its parity (study 1) or the font used to print the price stimulus (study 2).

The procedure consisted of two different phases: (1) the *evaluation phase and* (2) the *categorization* phase. In the *evaluation phase* (Fig 1A), participants learned a specific task-rule. The price of a series of product-price combinations had to be evaluated as ‘cheap’ or ‘expensive’ by pressing one of two keys as fast as possible. Here, it was important that the participants learned the task-rule required to evaluate prices as ‘cheap’ or ‘expensive’ (i.e. left key = cheap and right key = expensive).

The *evaluation phase* was followed by the *categorization phase* (Fig 1B). Again, participants observed a series of product-price combinations. However, participants no longer had to indicate whether they considered the price to be ‘cheap’ or ‘expensive’, but instead had to categorize the price based on the parity of the number (study 1) or the font used to print the price stimulus (study 2). The same keys as in the *evaluation phase* had to be used to respond in the *categorization phase*. Following the TRC logic, this allowed us to test whether the prices of the product were automatically evaluated. Namely, if the irrelevant task-rule (i.e. left key = cheap and left key = expensive) overlapped with the response needed to categorize the price stimulus, both responses were ‘congruent’ and faster response latencies were expected. However, if the valence of the irrelevant task-rule opposed the response required to categorize the price stimulus, both responses were ‘incongruent’ and slower response latencies were expected.

Crucially, the prices used during the *categorization phase* were manipulated to be perceived ambiguous (not evidently cheap or expensive) or unambiguous (evidently cheap or expensive). We assumed that unambiguous prices were unlikely to resemble participants’ internal reference price. In contrast, we assumed that ambiguous prices were likely to be arguably closer to the ‘true’ internal reference price. Therefore, it was expected that the size of the TRC effect would vary in accordance with the distance to the internal reference price of the participants. TRC effect were expected to be larger if the price was unambiguous (i.e. not close to the internal reference price). If the price was ambiguous, no clear TRC effects were expected. This is because the evaluation of a price stimulus as ‘cheap’ or ‘expensive’ should become fuzzier when the price approaches the internal reference price, leading to lesser activation of the irrelevant responses, resulting in smaller congruency effects. In this view, large TRC effects indicate that the price was distant from the internal reference price and small TRC effects indicate that the price was close to the internal reference price.

## Study 1

We wanted to test whether prices are evaluated automatically as ‘cheap’ or ‘expensive’. This automatic evaluation was expected to be reflected in a TRC effect between the *evaluation phase* and the *categorization phase*. Furthermore, we wanted to explore if the observed effects can be informative to estimate consumers’ WTP.

### Materials and methods

#### Ethical statement

Both studies were conducted in accordance with the ethical standards of the 1964 Declaration of Helsinki and approved by the rules of the Institutional Review Board from the Faculty of Psychology and Educational Science of Ghent University. All participants gave informed consent at the beginning of the experiment and were informed that participation was voluntary, and that all data would be processed and stored anonymously.

#### Participants

Sixty-one volunteers (M_age_ = 23.7 years old; 40 females) were recruited with the online recruitment platform of Ghent University and were paid €10 for their participation. All participants reported normal or corrected to normal vision.

#### Stimuli

Prior to the actual study, a pilot survey was conducted to test what products were suited for the study. Products selected for the study were known and valued by the test population (i.e. people active on the online recruitment platform of Ghent University). Furthermore, the target population was supposed to have an indication of the normal retail price of the products. To this end, members of the test population were asked if they were familiar with buying the product and to estimate the price the product. A total of 50 consumable products from renowned brands were included in the survey. Based on the collected data, 36 items were selected for the study.

In total, 28 products were presented during the *evaluation phase*. In order to facilitate the learning of the task-rule, an unambiguously cheap and unambiguously expensive price was created for each product. To create these unambiguous prices, current retail prices (i.e. average of the product’s current selling price in the big Belgian supermarkets) were decreased (or increased) by seventy percent (range unambiguous cheap price: [€ 0.25–€ 2.77]; range unambiguous expensive prices: [€ 2.43–€ 15.73]). This resulted in a total of 56 product-price combinations used in the *evaluation phase*.

During the *categorization phase*, eight other products were used as stimuli. Here, the price was varied across seven price levels, ranging from unambiguously cheap to unambiguously expensive prices. For each product, six prices were created based on the current retail price of the products. The retail price was manipulated in three steps. In a first step, the retail price was increased or decreased by 10%. It was expected that these prices would still be considered as ambiguous because we assumed that these prices would be more or less aligned with the internal reference price of the participants. In a second step, the retail price was increased or decreased by 40%. In the last step, the retail price was increased or decreased by 70%. It was expected that most participants would consider the prices in the 40%- and 70%-conditions as unambiguous. Finally, the retail prices (0% condition) were also included as price stimuli in the study. Retail prices were also expected to be perceived as ambiguous. Then, we created an additional group of prices by adding 0.01 euro to all prices. This created a stimulus set where there was an equal amount of even and an odd price for each price level. Finally, this resulted in a price spectrum (range of the used prices [€ 0.37–€ 6.36]) where the price varied across seven price levels. In total, 56 product-price combination were used during the *categorization* phase.

#### Procedure and task

All participants were invited to the laboratory, seated in front of a computer and received instructions on the screen of a computer. It was explained that the study consisted of two phases. First, in the *evaluation phase*, participants were instructed to press the F key with the left index finger to evaluate a product-price combination as ‘cheap’ and to press the J key with the right index finger to evaluate a product-price combination as ‘expensive’ (Fig 1A). Participants were encouraged to do this as fast as possible. In total, all 56 product-price combinations were presented twice in a random order. Resulting in a total of 112 trials in the *evaluation phase*.

Next, the *categorization phase* was initiated, and participants had to categorize prices based on the parity of the price stimulus. Participants had to use the F key to categorize a price as an even number (left-handed response = even number = previously linked to the cheap evaluation) and the J key to categorize a price as an odd number (Fig 1B; right-handed response = odd number = previously linked to the expensive evaluation). Thus, the task-rule learned in the previous phase became irrelevant. All 56 product-price combinations were presented randomly. Each product-price combination was shown exactly four times, twice with an even price and twice with an odd price. This resulted in a total of 224 categorization trials divided across two blocks; 112 even categorizations and 112 odd categorizations, balanced across price levels. To prevent that participants focused only on the numbers of the price stimuli and not on the link between the product and the price, refresher trials were added. On refresher trials a cue was used to instruct participants to evaluate the product-price combination as cheap or expensive. Participants were instructed to use the task-rule learned in the previous phase (i.e. F key = ‘cheap’ & J key = ‘expensive) to do this. In total, eight refresher trials were presented pseudorandomly, one refresher trial in each quarter of the block. Finally, after both response latency tasks, all product-price combinations used during the categorization phase were again randomly presented to the participants. Participants were asked to indicate whether they would buy the product for the given price. This allowed us to estimate buy-response functions. The total procedure lasted for approximately 30 minutes.

#### Stimulus presentation

The stimulus presentation was controlled by Tscope, a C library developed to program cognitive studies and to measure response latencies with millisecond accuracy [39]. Trials in the *evaluation phase* and in the *categorization phase* adopted the same sequence of events and only differed in the used stimulus set and the task participants were instructed to complete (Fig 1).

First, a fixation cross appeared on the screen for 1000 milliseconds. Then, the fixation cross was replaced by a product that stayed on the screen for 2500 milliseconds. Finally, the product was replaced by a price stimulus. The price remained on the screen until the participant responded. Upon the registration of the response, feedback was provided on the first 20 trials of each phase in order to reassure that participants understood and implemented the task correctly. If the participant responded accurate, the word “juist” (i.e. ‘correct’ in Dutch) was printed on the screen. If the response of the participant was wrong, the word “fout” (i.e. ‘wrong’ in Dutch) appeared. After 500 milliseconds, the feedback was removed from the screen and the next trial was initiated 500 milliseconds later. In trials where no feedback was provided, the inter trial interval was 500 milliseconds.

### Data analysis

#### Data cleaning and outlier removal

Both pre-processing and statistical analysis of the data was performed in R (R Development Core Team, 2013). Prior to the analysis, reaction time (RT) outliers (0.3%) were removed from the data because the responses were considered action slips (RTs < 100 ms) or inattentions of the participant (RTs > 4000 ms). After these outliers were excluded, RTs that deviated more than two standard deviations from the participants mean RT were also removed (4.9%). Data from the *evaluation phase* was used to identify participants with little or no notion about the value of the product-price combinations used in the study. An *evaluation response* was coded as an error [M_error_ = 7.4%, SD_error_ = 5.1] if the participant misevaluated an unambiguous price. Based on this reasoning, one participant was excluded from further analysis because the amount of erroneous responses made in the *evaluation phase* exceeded three times the standard deviation of the sample. One additional participant was excluded from further analysis because the amount of errors made in the *categorization phase* exceeded the average amount of errors [M_error_ = 5.5%, SD_error_ = 4.7] in the sample with three standard deviations. Here, an error was defined as wrongly categorizing the parity of the price stimuli. Overall, remaining erroneous responses made during the *categorization phase* were removed before further analysis was initiated.

#### Hypothesis testing

It was expected that the evaluation response would exert an automatic influence during the categorization phase. This was anticipated to result in a congruency effect between the relevant (i.e. parity categorization) and the irrelevant task-rules (i.e. price evaluation). To statistically account for both the participant and product random effects [40] a linear mixed model was fitted on the response latencies with *response* (left or right) as factor, *price level* (price level: -70%, -40%, -10%, 0%, +10%, +40% or +70%) as numerical predictor and the interaction term between both predictors [41]. The random effects structure was determined using a backwards selection procedure and consisted of a random intercept for participant and product and random slopes for *response* and *price level* [42]. Whereas the random effects structure allows to take in to account baseline differences in response latencies related to individual participants and products, the random slopes account for intra-individual differences related to the fixed effects structure of the model. Response latencies were defined as the time that elapsed between the presentation of the price stimulus and the categorization response of the participant. P-values were calculated on the basis of Satterthwaite approximated degrees of freedom.

#### Visualization and interpretation of the TRC effect

In order to interpret the congruency effect within the context of this study, it is important to remember that participants learned that left-handed responses were associated with cheap evaluations and right-handed responses with expensive evaluations. However, this task-rule became irrelevant and a new task-rule was learned during the *categorization phase*. Therefore, if the price was assumed to be perceived as ‘cheap’ by the participant and the required response was left (i.e. the price was an even number & previously learned ‘cheap evaluation’), the responses of both task-rules were considered as congruent. Also, if prices were assumed to be perceived as ‘expensive’ and required a right-handed response (i.e. the price was an odd number & previously learned ‘expensive evaluation’), both responses were considered congruent. Contrarily, if the price was assumed to be perceived as ‘cheap’ but required a right-handed response or the price was assumed to be perceived ‘expensive’ but required a left-handed response, the responses of both task-rules were considered incongruent. To visualize the task-rule congruency effect, the difference in response latencies between left-handed (i.e. previously learned ‘cheap evaluation’) and right-handed (i.e. previously learned ‘expensive evaluation’) responses was calculated for each price level individually. Knowing that difference scores above zero indicate that left-handed responses were faster than right-handed responses, positive difference scores indicate that the ‘irrelevant cheap evaluation’ facilitated the categorization response. Vice versa, difference scores below zero indicate that the ‘irrelevant expensive evaluation’ facilitated the categorization response. However, if the difference score is zero, this suggests that none of irrelevant responses (i.e. cheap or expensive) had a profound influence on the categorization response.

### Results

#### Evaluation phase

In total, 92.8% of the evaluations in the cheap condition and 91.9% of the evaluations in the expensive condition were evaluated correctly. This shows that participants included in our analysis were able to distinguish unambiguously cheap from unambiguously expensive prices.

#### TRC effect

**Fig 2:**
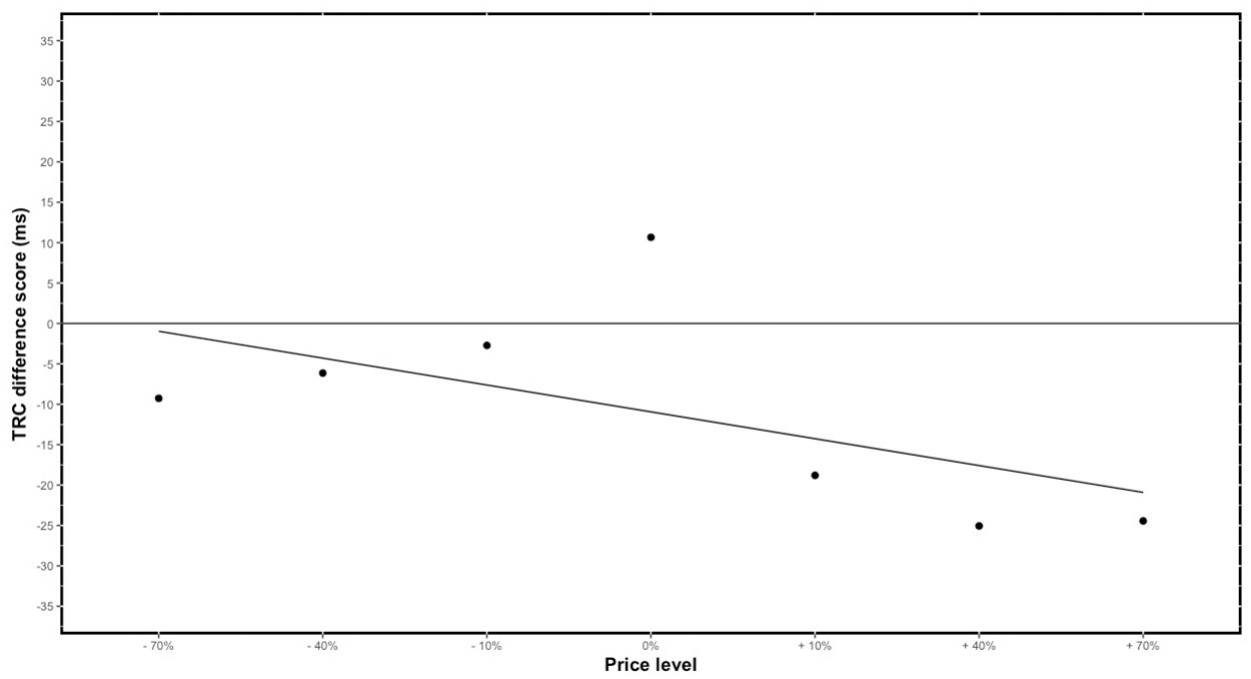
Study 1—Visualization of the TRC effect: Observed data vs. model predictions. Difference scores were calculated between response latencies of right- and left-handed responses (RT left minus RT right) for each price condition. The average difference scores per price condition are represented by the points on the graph. While the line on the graph represents the prediction of the mixed model.

The main effects of *response* [t(44.8) = .11, β = .48, p = .91] and *price level* [t(9.29) = 1.45, β = 2.0, p = .18] were not significant. Importantly, the interaction between *response* and *price level* was significant [t(11764) = 1.98, β = 1.66, p < .05]. This indicates that the irrelevant task-rule (i.e. price evaluation) influenced the categorization response in a price dependent manner. As can be seen on Fig 2, there are clear congruency effects observed in the upward price manipulations expensive conditions (plus 40% and plus 70%). This suggests that categorization responses were influenced by the irrelevant response in these price conditions. Namely, that irrelevant expensive responses were faster than irrelevant cheap responses in the conditions where the price was increased.

### Discussion

We provide evidence for a TRC effect between the *evaluation phase* and the *categorization phase* of the study. More concretely, we provide evidence for the notion that prices are evaluated automatically within the outlined procedure.

The observed interaction between *response (i*.*e*. left or right) and the absolute value of the price stimulus shows that the TRC effect depends on the price manipulation. Opposed to what was expected the difference scores all calculated difference scores, other than the 0% price condition, were negative. The congruency effects only showed the anticipated effect in the *plus 40%* and *plus 70%* conditions. Based on the data of this study we only provide evidence for the notion that unambiguous expensive prices are automatically evaluated as expensive. Furthermore, all difference scores of the price conditions where prices were manipulated to be evaluated as cheap, are below zero, which would indicate that all prices were implicitly evaluated as expensive. Because the pattern of the TRC effect does not follow the expected results, an optimization of the procedure was required in order to enable implicit WTP estimates.

Taking into account the results if this study, it is likely that the categorization task was not optimal for our purpose because participants might have adopted a particular strategy in order to perform optimal in the *categorization phase*. Even though catch trials were introduced to force the participants to link the product to the price, they might have only used the last digit of the price stimulus to resolve the parity categorization task. Still, the interaction between response and price level shows that the price evaluations influenced categorization responses, while this was irrelevant for the task at hand.

Taken together, we provide evidence that the used procedure taps in to the cognitive processes underlying price evaluations. However, the choice for parity categorization as secondary task seemed to be a confounding factor in our study, possibly masking the effect that we intended to measure. This problem is addressed in the next study.

## Study 2

Again, the goal of the study was to determine if prices were evaluated automatically as cheap or expensive within the proposed procedure. Based on the results of study 1, a new task was introduced during the *categorization phase*. Instead of categorizing the prices based on the parity, participants now had to categorize based on the font. It was expected that this would result in a clearer measure of the cognitive processes involved during price evaluation.

### Materials and methods

#### Participants

Sixty-two volunteers (M_age_ = 22 years old; 47 females) were recruited with the online recruitment platform of Ghent University were paid €10 for their participation.

#### Material, procedure and task

Both the products and the price stimuli used in the study were identical to the first study. All participants were invited to the laboratory, seated in front of a computer and received instructions on the screen of a computer. The task-rule in the evaluation phase was the same as in the first study (i.e. press the F key if the price is cheap; press the J key if the price is expensive). Different than in the first study, participants were now instructed to categorize price stimuli based on the font of the price stimulus (i.e. regular or italic font). It was assumed that this task would stimulate participants to process the price stimulus in a holistic manner. Furthermore, we presumed that attention would be directed toward the price as a stimulus rather than to the last digit. Also, we expected that less cognitive resources would be needed to complete this task, compared to the parity task, since participants were not necessarily required to process the number before responding.

In the *categorization phase*, the font of the price stimulus was balanced across price conditions and determined at random on a trial-by-trial basis. The task-rule used during the *categorization phase* was counterbalanced between participants. One half of the participants had to indicate that price was printed in regular font with a left-handed response (i.e. F key) and that price was printed in italic font with a right-handed response (i.e. J key), the other half of the sample was instructed the opposite. This does not influence the interpretation of the results. Our hypothesis is based on the transfer of the task-rule learned in the *evaluation phase* to the *categorization phase*, which is independent of the task in the *categorization phase*. Other than this, the study was identical to the first study (Fig 1).

#### Data analysis

**Data cleaning and outlier removal.** Both the pre-processing and the statistical analysis were the same as described in study 1. Again, reaction time (RT) outliers (0.9%) were removed from the data prior to the analysis because the responses were considered action slips (RTs < 100 ms) or inattentions of the participant (RTs > 4000 ms). To further account for outliers, RTs that deviated more than two standard deviations from the participants mean were also removed (4.8%). One participant was excluded from further analyses because the amount of errors made in the *evaluation phase* exceeded three times the standard deviation of the sample [M_error_ = 8.8%, SD_error_ = 4.8]. One additional participant was excluded from further analyses because the amount of errors made in the *categorization phase* exceeded the average amount of errors in the sample by three standard deviations [M_error_ = 4.2%, SD_error_ = 3.3]. Overall, erroneous responses made during the *categorization phase* were removed before further analysis was initiated. The remaining analyses were identical to the procedure described in the first study.

**Hypothesis testing.** Again, a linear mixed model was fitted on the response latencies with *response* (left or right) as factor, price level (price level: -70%, -40%, -10%, 0%, +10%, +40% or +70%) as numerical predictor and the interaction term between both predictors [41]. The random effects structure and all other statistical tests were identical as reported in study one.

**Visualization and interpretation of the TRC effect.** Again, it is important to define what congruency means within the context of this study in order to interpret the results. Knowing that participants learned a task-rule were left-handed responses were associated with cheap evaluations and right-handed responses with expensive evaluations and, that a new task-rule was introduced in the categorization phase, the same reasoning can be followed as explained in the first study. If prices were assumed to be perceived as ‘cheap’ and a left-handed response was required (the price was printed in either regular/italic font & previously learned ‘cheap evaluation’) or if prices were assumed to be perceived as ‘expensive’ and a right-handed response was required (the price was printed in either regular/italic font & previously learned ‘expensive evaluation’), then both task-rules were considered as congruent. Vice versa, if prices were assumed to be perceived as ‘cheap’ and a right-handed response was required or prices were assumed to be perceived as ‘expensive’ and a left-handed response was required, then both task-rules were considered incongruent. Similar as in the first study, difference scores were calculated between response latencies of right- and left-handed responses (RT left minus RT right) for each price condition. Difference scores above zero indicate that left-handed responses were faster than right-handed responses, positive difference scores therefore indicate that the ‘irrelevant cheap evaluation’ facilitated the categorization response. And difference scores below zero indicate that the ‘irrelevant expensive evaluation’ facilitated the categorization response, while a difference score of ‘zero’ indicates that there was no difference between both irrelevant responses.

### Results

#### Evaluation phase

In total, 92.2% of the evaluations in the unambiguously cheap condition and 90.2% of the evaluations in the unambiguously expensive condition were correct. This indicates that participants included in the analysis were able to differentiate between cheap and expensive prices.

#### Influence of the irrelevant response

**Fig 3:**
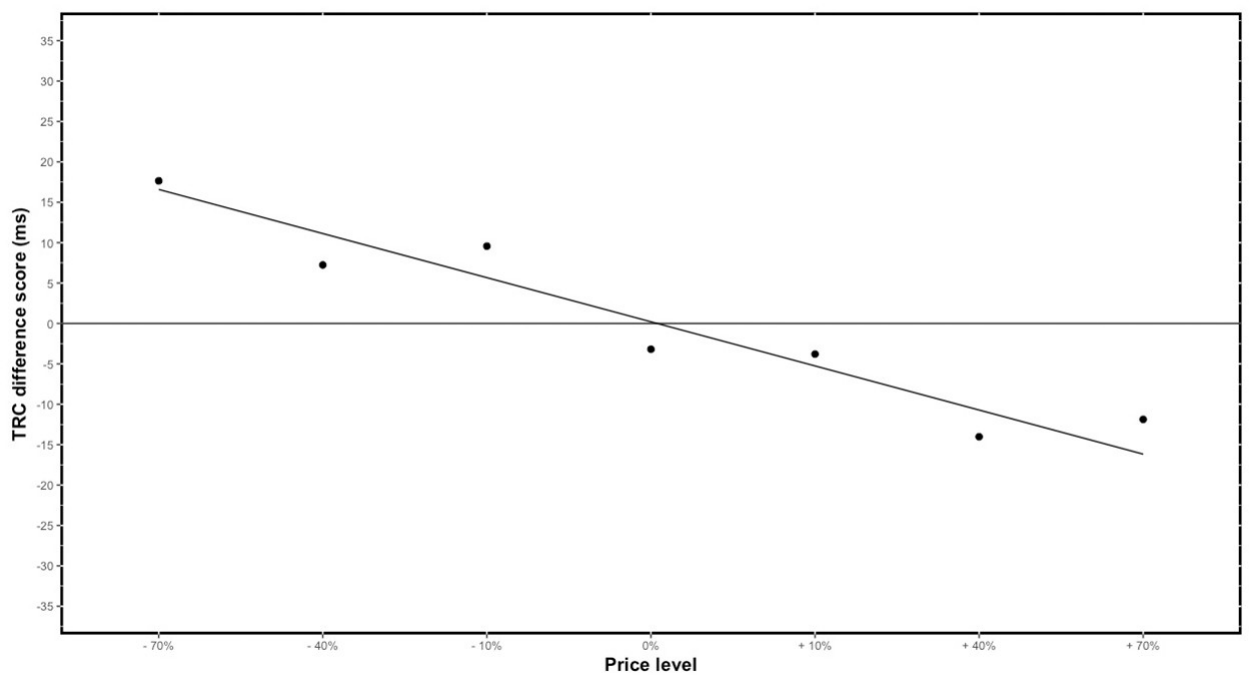
Study 2—Visualization of the TRC effect: Observed data vs. model predictions. Differences scores were calculated between response latencies of right- and left-handed responses (RT left minus RT right) for each price condition. The average difference scores per price condition are represented by the points on the graph. While the line on the graph represents the prediction of the mixed model.

The main effect of price level was not significant [t(7.6) = 1.46, β = 1.84, p = .18]. Yet, the main effect of *response* was significant [t(82.2) = -2.1, β = -8.3, p < .05], indicating that left-handed responses were on average faster than right-handed responses. Again, the interaction between price level and *evaluation response* was significant [t(12113) = 3.7, β = 2.7, p < .001], suggesting a TRC effect. This provides evidence for the notion that the irrelevant task-rule (i.e. price evaluation) influenced the categorization response in automatically and that this effect is dependent on the price condition. Also, the congruency effect followed the expected pattern (Fig 3) and can therefore possibly be used to estimate consumers’ WTP. The TRC effect had the highest positive value in the unambiguous cheap conditions (minus 70%) and the most negative values in the unambiguous expensive conditions (plus 70% and plus 40%). Also, the TRC effect varied around zero for the ambiguous price conditions (retail and minus 10%). This pattern indicates that very cheap prices were automatically evaluated as “cheap” and that very expensive prices were automatically evaluated as “expensive”. Whilst ambiguous prices were neither automatically evaluated as “cheap” or “expensive”.

### Discussion

Based on the results of the first study, the task used during the *categorization phase* of study 2 was adjusted. Participants were now instructed to categorize price stimuli based on the font of the price stimulus (i.e. regular or italic print). We reasoned that this task would interfere less with the assumed automatic evaluation process and that this would therefore provide a clearer measure of the underlying cognitive processes. The results of the study further strengthened the notion that price stimuli are evaluated automatically. Furthermore, the observed TRC pattern allows to further explore if the outlined procedure can be used to estimate consumers’ WTP. In order to illustrate how outlined procedure and the TRC effect can be used to derive WTP estimates we explain how the data pattern of the current study can be used to guide WTP estimates.

### Interpretation of WTP and relationship with buy-response function

**Fig 4:**
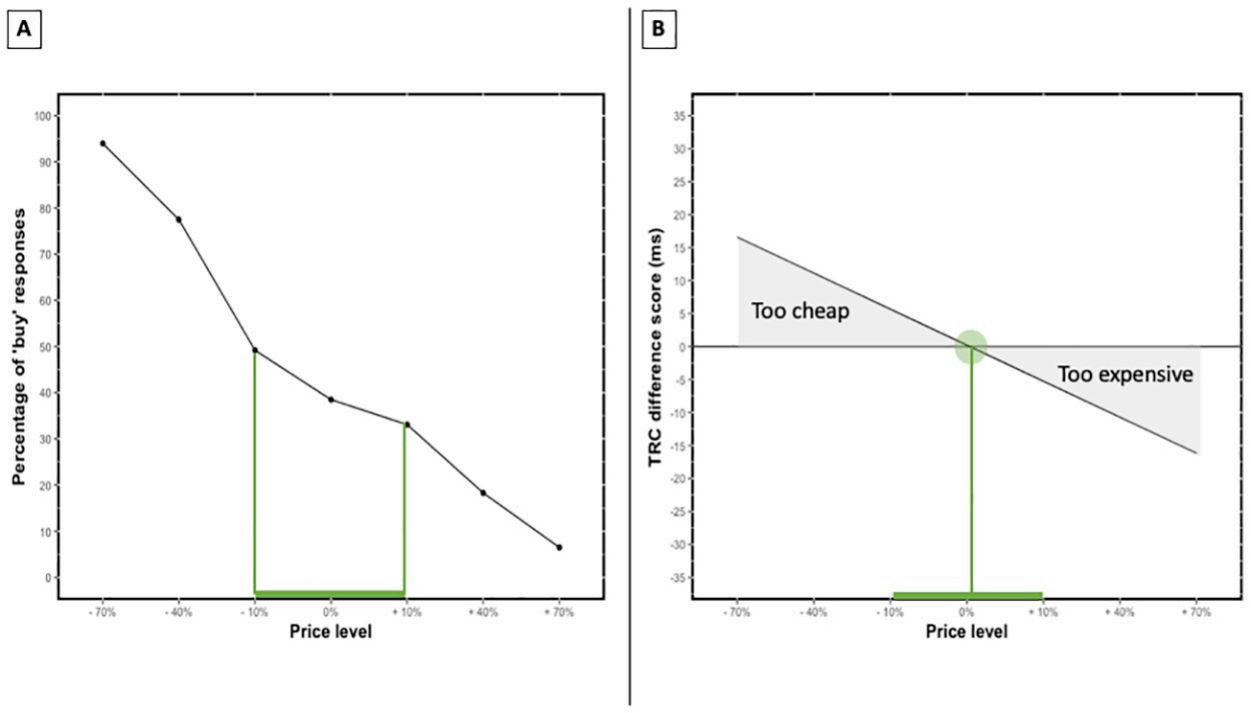
Using model predictions to estimate consumers’ WTP. (A) The buy-response function estimated on the group level at the end of study two. This function indicate what percentage of the sample was willing to buy the product at the given price. (B) Regression line estimated by the mixed model fitted on the RTs of study two. Values that are lower than zero can be considered as ‘too cheap’ while values above zero can be considered ‘too expensive’. The point where the regression line crosses the origin can be considered as the ‘optimal price point’.

Firstly, it is important to note that the procedure presented in this paper aims to determine consumers’ WTP for marketable goods (e.g. FMCG goods, electronics,). More complex subjective value taxations, such as these of non-marketable goods (e.g. clean tap drinking water), cannot be addressed with this method. In order to estimate the WTP for these daily consumer goods, the regression line estimated by the mixed model is used as the starting point. The size of the TRC effect clearly show a linear decrease (Fig 3). Knowing that difference scores above zero represent ‘automatic cheap’ evaluations and difference scores below zero represent ‘automatic expensive’ evaluations, the regression line can be used to guide WTP estimates. Namely, the point where the regression line crosses the origin can be interpreted as the ‘optimal price point’. In this price condition, the price is neither evaluated as ‘cheap’ or ‘expensive’. It is therefore likely that this price point is closely related to the internal reference price and consequentially to the WTP of the participants [26–28]. According to the data of study two (Fig 3), the best price is slightly higher than the retail price (Fig 4B). Moreover, all prices where the regression line has a value above zero can be interpreted as ‘too cheap’ and all prices where the regression line values are below zero can be interpreted as ‘too expensive’. In both instances one of the responses related to the irrelevant task-rule (i.e. ‘cheap’ or ‘expensive’ evaluation) had a profound influence on the relevant response.

Moreover, the implicit estimate that results out of current study relates to the buy-response function estimated at the end of the study (Fig 4A). This is not surprising since both the explicit buy-response function and the implicit WTP estimates are expected to be related to each other as both measures to build on consumers’ WTP. Still, two interpretations can carefully be derived from the buy-response function. First, there is a linear decrease of the buy-response function in accordance with the price of the product. Participants were less prone to buy the product as the price increased. A similar pattern emerged in the data of study two. Second, the number of participants that indicated to buy the product decreased faster in the unambiguous price conditions. For the ambiguous prices there was no such radical decline in buy-responses, indicating that the optimal price is likely to be located in one of these price conditions (minus 10%, retail and plus 10%). However, this interval consists out of multiple price conditions and therefore there is no conclusive answer on what the ‘optimal price point’ is. We suggest using the information gathered with an implicit procedure to pin-point the optimal price within this interval (Fig 4B). Here, the ‘optimal price point’ is located within this interval, which indicates that the implicit procedure relates to the stated purchase intentions of the participants which makes it plausible that the procedure is actually tapping in to the ‘true’ WTP of consumers. Yet, it is important to note that this interpretation outlines the conceptual framework that can potentially be used to gauge consumers’ WTP. This ‘proof-of-principle’ study is therefore intending to stimulate future research that fine grains and validates the implicit WTP estimation procedure.

## General discussion

The current paper sought to investigate if consumers automatically evaluate prices and additionally explored if these automatic evaluations can be used to implicitly estimate consumers’ WTP. To this end, we studied the TRC effect [34–37]. Within cognitive psychology numerous studies that used the TRC effect have demonstrated that irrelevant task-rules can influence cognitive processing on a second task. Because TRC effects are the result of automatic processes [35], the reported effects are considered as immediate, unintentional and thus implicit price evaluations [23,24,43]. It was investigated to what extent the TRC effect can be used to estimate consumers’ WTP. Finally, a conceptual illustration demonstrated how the procedure can be used to implicitly estimate consumers’ WTP. Hence, the contributions of this paper are both theoretical and applied. Theoretical because this is the first study providing evidence for the notion that prices can be evaluated without deliberate processing. And applied because an important problem of the current indirect and direct WTP estimates is addressed in this paper: explicit answering biases. While the procedures used to day all rely on explicit consumer responses related to the (price of the) product, the procedure outlined in this paper is the first to provide the opportunity to estimate WTP without asking questions related to product or its price. Nonetheless, more research is needed to investigate the validity of the outlined procedure.

In two studies, evidence was presented in favor of the notion that participants evaluate price stimuli automatically. Namely, in both studies TRC effects were observed between irrelevant price evaluations and task relevant responses, suggesting that the irrelevant task-rule (i.e. ‘cheap’ or ‘expensive’ evaluations) influenced cognitive processing during categorization based on the parity (study 1) or font (study 2) of the price stimuli. Following the TRC logic, this implies that prices are implicitly evaluated as ‘cheap’ or ‘expensive’. But how can these automatic effects be used to estimate consumers’ WTP? Since the absolute value of the used prices was manipulated in order to deviate from the internal reference prices of the participants, we were able to put forward a clear hypothesis: the absolute size of the TRC effect should vary according to the absolute size of the price stimulus. That is, unambiguous ‘cheap’ and ‘expensive’ prices were expected to result in the largest TRC effects. In contrast, ambiguous prices were expected to be less susceptible to TRC effects, because they were expected to be perceived as neither evidently ‘cheap’ nor evidently ‘expensive’. In study 2 (font categorization) we provided evidence for this hypothesis. In the section above it was illustrated how this variable TRC effect can be used to guide estimates of consumers’ WTP. Yet, these studies can be considered as a first but important step towards an implicit estimate of consumers’ WTP. More work needs to be done before this procedure can be applied in the real world.

### Short comings and future research

The idea to use the TRC effect as an implicit estimate of consumers’ WTP is new. Forasmuch as our intention was to demonstrate that the TRC effect can possibly be used to determine consumers’ WTP, we opted to conduct research in the laboratory because this allows a maximum of experimental control. In order to make the procedure suited to estimate consumers’ WTP for real products, future research needs to address three important problems: (1) the time needed to complete the study needs to be limited, (2) the design of the experiment needs to be reconfigured and, most importantly, (3) the WTP estimates generated by the procedure need to be validated.

In order to obtain sufficient statistical power to address the research questions a within-subjects design was used. While the experiments illustrated that TRC effects elicited in the presented procedure can be used to implicitly determine WTP, the within-subjects experimental design does not allow us to put forward estimates regarding individual products. That is, all product-price combinations were presented multiple times to the participants and then the response latencies across price conditions were compared. While we addressed the repeated-measures problem with mixed model analysis, future research could address this problem by setting up a between-subjects design. Then, each participant would only be exposed once to each product-price combination, making the procedure more ecologically valid and shorter. However, depending on the particular research question, a between-subjects design would require significantly more participants to make valid statistical inferences [44]. Hence, it would be practical to conduct this type of studies via the world wide web. Recent developments in online psychological research show that it is relatively simple to program and conduct reliable cognitive studies online [45–47]. Additionally, conducting online studies would allow researchers to target specific segments of ‘real’ consumers, which would further increase the ecological validity of the procedure. Taken together, we consider shortening the procedure and, as such making the procedure more user friendly, one of the key challenges for future research.

In this paper, large price manipulations were knowingly used in order to create a context where price could be evaluated as unambiguous cheap or expensive. However, as this methodology evolves to more applied contexts, smaller price manipulations will have to be used (e.g. -15%, -10% -5%, +5%, +10% and +15%) in order obtain valuable WTP estimates. Here, the main question would be if the TRC effect shows the same pattern if the price manipulations are more conservative. Yet, the results of the second study show that there is variation in the TRC effect around the ambiguous price conditions.

Future studies need to investigate if the proposed methodology actually results in valid WTP estimates. Most importantly, we assumed that the internal reference price of the participants would be closely related to the current market price (i.e. retail price). It is likely that the internal reference standards differ between participants, or even within a single participant depending on the purchase context (e.g. buying a soda in the airport or in the supermarket might yield in different reference standards). Yet, the results of the second study seem to indicate that the internal reference price relates to the market price. Nevertheless, future research needs to investigate whether the proposed WTP estimation procedure can be applied on the individual level and whether particular characteristics or contexts have a profound impact on the results. Furthermore, we assumed that participants used this internal reference price as a standard to evaluate the prices during the studies as “cheap” or “expensive”. However, it is possible that other processes are driving the effect that we measure. To exclude alternative interpretations future research should compare the estimates resulting from the proposed method with other methods used to estimate consumers’ WTP [10] or even relate the estimates to actual sales data. Furthermore, reliable WTP estimates would allow companies to adopt pricing strategies constructed around consumers’ WTP. An approach that has been proven to be more effective and therefore yield competitive advantages [48–51].

Other than the practical application, this study also contributes theoretically to the field of behavioural price research. This vein of the pricing literature investigates how consumers perceive, process and evaluate price stimuli [19,52–55]. In a recent review article of the field, it was pointed out that more research needs to focus on understanding how people process price stimuli non-deliberately [56] and to understand the relative ease or difficulty at which different price stimuli are perceived and processed [57]. Since the presented procedure allows researchers to measure ongoing cognitive processes, rather than retrospective interpretations of these processes [58], it can be used to address fundamental questions regarding the processing of price stimuli.

In conclusion, we presented two studies that indicate that prices are evaluated automatically as “cheap” or “expensive”. It was explored whether the underlying cognitive processes can be used to estimate consumers’ WTP. Then, it was illustrated how information regarding consumers’ WTP can be obtained implicitly. While the results of presented in this paper are promising more research needs to be done in order to determine the validity of the procedure and to translate this implicit methodology to marketing practice.
